# Genome-wide copy number variant analysis for congenital ventricular septal defects in Chinese Han population

**DOI:** 10.1186/s12920-015-0163-4

**Published:** 2016-01-08

**Authors:** Yu An, Wenyuan Duan, Guoying Huang, Xiaoli Chen, Li Li, Chenxia Nie, Jia Hou, Yonghao Gui, Yiming Wu, Feng Zhang, Yiping Shen, Bailin Wu, Hongyan Wang

**Affiliations:** 1 Institutes of Biomedical Sciences and Children’s Hospital, Fudan University, 131 Dongan Road, Shanghai, 200032 China; 2 The State Key Laboratory of Genetic Engineering, Ministry of Education (MOE) Key Laboratory of Contemporary Anthropology, and Collaborative Innovation Center of Genetics and Development, School of Life Sciences, Fudan University, Shanghai, 200433 China; 3 Jinan Military General Hospital, Jinan, 250022 China; 4 Children’s Hospital, Fudan University, Shanghai, 201102 China; 5 Capital Institute of Pediatrics, Beijing, 100020 China; 6 Department of Biology, Changzhi MedicalCollege, Changzhi, 046000 China; 7 Department of Laboratory Medicine, Children’s Hospital Boston, Harvard Medical School, Boston, MA 02453 USA; 8 School of Life Sciences, Obstetrics & Gynecology Hospital, Institute of Reproduction & Development, Fudan University, Shanghai, 200032 China

**Keywords:** Ventricular septal defect, aCGH, Congenital heart defect, Copy number variants

## Abstract

**Background:**

Ventricular septal defects (VSDs) constitute the most prevalent congenital heart disease (CHD), occurs either in isolation (isolated VSD) or in combination with other cardiac defects (complex VSD). Copy number variation (CNV) has been highlighted as a possible contributing factor to the etiology of many congenital diseases. However, little is known concerning the involvement of CNVs in either isolated or complex VSDs.

**Methods:**

We analyzed 154 unrelated Chinese individuals with VSD by chromosomal microarray analysis. The subjects were recruited from four hospitals across China. Each case underwent clinical assessment to define the type of VSD, either isolated or complex VSD. CNVs detected were categorized into syndrom related CNVs, recurrent CNVs and rare CNVs. Genes encompassed by the CNVs were analyzed using enrichment and pathway analysis.

**Results:**

Among 154 probands, we identified 29 rare CNVs in 26 VSD patients (16.9 %, 26/154) and 8 syndrome-related CNVs in 8 VSD patients (5.2 %, 8/154). 12 of the detected 29 rare CNVs (41.3 %) were recurrently reported in DECIPHER or ISCA database as associated with either VSD or general heart disease. Fifteen genes (5 %, 15/285) within CNVs were associated with a broad spectrum of complicated CHD. Among these15 genes, 7 genes were in “abnormal interventricular septum morphology” derived from the MGI (mouse genome informatics) database, and nine genes were associated with cardiovascular system development (GO:0072538).We also found that these VSD-related candidate genes are enriched in chromatin binding and transcription regulation, which are the biological processes underlying heart development.

**Conclusions:**

Our study demonstrates the potential clinical diagnostic utility of genomic imbalance profiling in VSD patients. Additionally, gene enrichment and pathway analysis helped us to implicate VSD related candidate genes.

**Electronic supplementary material:**

The online version of this article (doi:10.1186/s12920-015-0163-4) contains supplementary material, which is available to authorized users.

## Background

Congenital heart defects (CHDs) are the most prominent birth defects, with a prevalence of 4 to 10 per 1000 live births [1]. A ventricular septal defect (VSD) occurs in more than 1 in 300 live births and is the most common CHD identified to date [2]. Although nearly 40 % of infants with VSDs can survive without treatment up to the age of 15 years, VSD patients diagnosed in adulthood may experience potentially serious clinical and hemodynamic problems [3]. Early detection and diagnosis lead to improved prognosis for patients with CHD.

Genomic imbalances detected by karyotype or FISH explain 9 % to 18 % of neonatal CHD cases [4]. CHD-related CNVs, identified by chromosomal microarray analysis (CMA), have been reported on almost every human chromosome [5–9] and numerical chromosomal abnormalities such as trisomy 21, trisomy 18 and trisomy 13 and also CNVs such as 22q11.2 deletion are causally related to CHD. Although the causal relationship between CNVs within the size range of 100 kb-1 Mb and CHD is incompletely investigated, rare *de novo* CNVs were revealed up to 5 % of CHD trios [10].

Some CNV studies focus on one type of CHD such as syndromic CHD[5], tetralogy of Fallot[8], double outlet right ventricle[11], thoracic aortic aneurysms and dissections[12] and isolated congenital heart disease[9]. Aproximately 10 % of Tetralogy of Fallot CHD patients (TOF) display an increased genome-wide CNV burden [8, 10]. Hence,while Studies focusing on the involvement of CNV in CHD development have been reported [5, 7, 8, 12], the complex and heterogeneous phenotypic and genetic nature of CHD suggest the need for further investigation of their genetic basis, particularly for certain category of CHD.

The aim of the present study was to detect CHD-associated CNVs in Chinese patients with VSD. Although several studies had examined the occurrence of CNVs in Chinese CHD patients [13, 14], the CNVs in the Chinese patients with VSD have not been particularly investigated. Detecting the CNVs in patients with VSD may reveal VSD specific candidate genes and associated pathways.

## Methods

### Subjects

The subjects were recruited from multi-center hospital-based CHD cohort between 2000 and 2009. We randomly enrolled 166 unrelated patients (Subject details in Additional file 1: Table S1). All patients except seven had VSD phenotype. Every subject underwent complete cardiac evaluation. Congenital cardiac malformations were diagnosed by echocardiography and subsequently confirmed during surgery when performed. We categorized cases into two large groups: Isolated VSD (patients with VSD as the only cardiac defect) and complex VSD (patients with more than two additional cardiac phenotypes besides VSD). The additional phenotype besides cardiac phenotype such as mental defect or developmental disability was not discussed due to lack of clinical evaluation. The ethics committee of Fudan University approved the study. Documented consents were obtained from all participating patients or their legal guardians.

### CNV callings and rare CNVs identification

The Agilent Human Genome CGH microarray 244 k kit was used for CMA analysis (Agilent Technologies). Sample-specific CNV regions were identified using two software packages, Agilent DNA Analytics 4.0 CH3 Module (Agilent Technologies) and Nexus Copy Number v5.0 (BioDiscovery). Copy number gains or losses identified by both software packages were further manually inspected and confirmed.

**Fig. 1:**
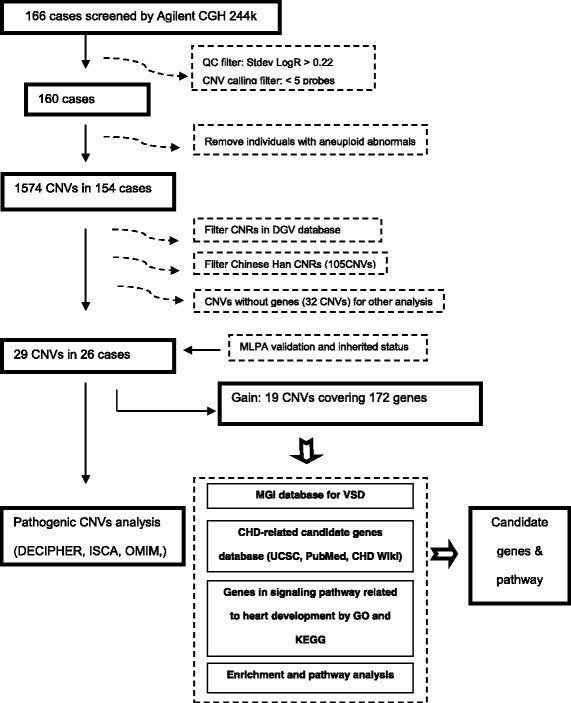
Workflow of CNV analysis and candidate genes discovery. CNV calls by DNA Analytics were performed by using the ADM2 algorithm, with a sensitivity threshold of 6.0 and a minimum of 5 probes. The QC metrics table was used to check signal intensity and background noise. Above 0.22 of DLR Score (Derivative Log Ratio) was set as the cutoff to avoid false CNVs. 6 cases were removed because of bad quality of data during the QC filter.6 cases with aneuploid abnormals (Trisomy X and Down syndrome) were not put into further analysis

We interpreted the CNVs hierarchically as shown in Figure 1. Common CNVs were removed based upon their frequency in DGV (Database of Genomic Variants) [15, 16] and Chinese control data sets which were compiled from four published data sets including 10 individuals from Park et al. [17], 779 individuals from Lin et al. [18], 99 individuals established by SGVP (Singapore Genome Variation Project) [19] and 80 Han Chinese by Lou et al. [20]. CNVs with >70 % overlap with the ones reported in DGV were considered as common CNVs; CNVs partially (< 30 %) overlapped or with no overlap with the DGV dataset or other data sets were considered as rare CNVs. For the rare CNVs, we consulted the DECIPHER (https://decipher.sanger.ac.uk/) and ISCA (now as Clingene, https://www.clinicalgenome.org/) databases for evidence of clinical relevance [21]. The Refseq genes which included in CNVs were identified by UCSC browser (Human NCBI36/hg18 Assembly).

### Validation of small rare CNVs

CNVs with marginal QC values or of small size (< 80 kb) were selected to be confirmed by multiplex ligation-dependent probe amplification analysis (MLPA) (MLPA probes are listed in Additional file 1: Table S2). We also performed parental testing for 16 probands as listed in Additional file 1: Tables S3–S4.

### Statistical analyses

Statistical analysis was performed using SPSS 17. Two-side Fisher’s exact test and Student’s *t*-test were performed for qualitative and quantitative variables respectively.

### Identifying CHD-associated genes

In order to identify VSD related genes, we compared the genes located in our rare CNVs with known CHD candidate genes. The Mouse Genome Informatics resource (MGI, http://www.informatics.jax.org/) can be very informative for studying disease-related genes in the human. We used “abnormal interventricular septum morphology” as the MP term to search for VSD related genes listed in MGI (MP: 0000281 as shown in the Additional file 1: Figure S1; http://www.informatics.jax.org/) and identified 147 genes with 375 genotypes and 416 annotations from MGI. In addition, 202 CHD-related genes were compiled from other resources: 104 genes from UCSC with the Human Genome Build 19 (cardiac gene: 76, cardiac transcription factor gene: 28), 51 genes from published literature (non-syndromic and syndromic CHD) and 47 genes from the CHD wiki. We also collected gene sets from the term “cardiovascular system development” (GO: 0072358) and candidate pathways involved in cardiac development such as Wnt, Notch, Hedgehog and FGF by KEGG and Netpath (http://www.netpath.org/). The CHD-related pathway selection processes are listed in Additional file 1: Figure S2. In total, there are a total of 1957 collected genes involved in cardiac related pathways which were combined as a potentially CHD- related dataset for further analysis. We compared the above combined data sets with genes mapping to CNVs detected in VSD patients.

### VSD candidate gene identification and pathway analysis

To define the most promising candidate genes from above defined gene list, ToppGENE was used as a gene prioritization and enrichment tool [22]. We used Ingenuity Pathway Analysis (IPA) to annotate genes encompassed within VSD-related CNVs for their molecular and cellular functions and associated pathways. Network scores were calculated based on the hypergeometric distribution and Fisher’s exact test.

## Results

### Chromosomal imbalances in VSD patients

We identified six aneuploid abnormalities: two cases of trisomy X (47, XXX) and four of trisomy 21. Up to 70 % of Down syndrome [23]subjects but only 1.3 % of trisomy X subjects have been reported to display CHD. CHD features in the trisomy X patients included VSD, ASD (Atrial septal defect), pulmonic and aortic stenosis coarctation [24].

### Rare CNVs in VSD patients

There were 1575 CNVs detected in our 154 patient cohort, with a median size of 310.5 kb (max 33.4 Mb, min 6.7 kb), compared with a median size was 52.9 kb (max 2.1 Mb, min 1.1 kb) in 965 controls. We identified 29 rare genic CNVs (CNV with at leaset one gene mapping to the dosage altered region ) in 25 of 153 VSD cases (16.3 %). Nighteen duplication CNVs involved 172 genes while 10 deletion CNVs affected 113 genes (Fig. 1). We also detected 32 intergenic CNVs but these were excluded from further analysis (Additional file 1: Table S9). 24.1 % (7/29) of the genic CNVs were less than 100 kb, 34.4 % (10/29) were from 100 kb to 500 kb and 41.3 % (12/29) were larger than 500 kb as shown in Additional file 1: Figure S3. Parental testing revealed that six CNVs were inherited from unaffected parents, reducing the likelihood that these are clinically significant. Three CNVs were confirmed as *de novo*: one deletion of 57.9 kb at Xp22.2 involving *EGFL6* gene (Additional file 1: Figure S4) and two duplications of 156.0 kb at 14q32.12, and of 117.8 kb in 7p14.2, which were experimentally confirmed; the two CNV gains were found in same subject.

### CNVs larger than 1 Mb

Five VSD cases revealed CNVs larger than 1 Mb (as shown Additional file 1: Table S5). Case 1 and 2 (NS255 and NS7783) had deletion at 22q11.2 (DiGeorge Syndrome). Case 3 (NB245) carried an 11.9 Mb duplication at 16p13.11-p11.2 involving 105 genes. This duplication was detected in a neonate with VSD, ASD and unexplained seizure. Case 4 (NA082) had a 10 Mb deletion at 4q34.3-q35.1 and a 18 Mb duplication at 3q26.32-q29. Both termed 4q loss and 4q syndrome are known to be associated with cardiovascular abnormalities [25]. Case 5 (NA252) had a 1 Mb loss at Xq21.1 and deletion at Xq21.1-q21.31 has been reported in patients with cardiac and renal anomalies [26]. The Xq21.1 deletion region encompasses the genes *CYSLTR1, GPR174, LPAR4, MIR4328, P2RY10* and *ZCCHC5.*

### CNVs putatively associated with VSD

All of the identified 29 rare CNVs, putatively causally associated with VSDs were placed on the chromosomal map of the genome (Additional file 1: Figure S5). These CNVs comprised mostly subtelomeric or centromeric imbalances and distributed on chromosomes such as 2p, 2q, 3p, 4q, 6p, 15q, 16q, 21q and 22q and most of these CNVs located on chromosomes 2, 3, 4, 7, 16 and X. The CNVs sizes identified in our VSD study are much smaller than those deposited in CHD wiki, which reports three regions (4q-ter, 15q26.2, 16q22) and one gene (*TBX1*) related to CHD. Twelve of the 29 CNVs (41.3 %) affect regions known to be ASD, VSD or general heart disease-related in DECIPHER and ISCA (Table 1).

**Table 1:** Summary of rare CNVs identified in CHD patients include genes

sample ID	Gender	age	Cytoband	Chromosome Region	Event	Interval(kb)	Count of Gene	Major candidate genes^a^	CHD phenotype	DECIPHER/ISCA/OMIM (no. of clinical features)^a^
NA012	F	1y8m	6p12.1	Chr6:55,823,840-55,949,133	Gain	125	1	*BMP5*	VSD	/
NA027	M	1y3m	2q35	Chr2:216,757,058-216,797,948	Gain	41	1	*XRCC5*	VSD	/
NA067	M	7 m	4q12	Chr4:57,627,423-57,940,932	Gain	314	2	*IGFBP7, LOC255130*	VSD	/
NA068	F	5y5 m	16q22.1	chr16:67,868,480-68,086,257	Loss	218	8	*COG8, CYB5B, NIP7, PDF, SNTB2, TERF2, TMED6, VPS4A*	TGA	VSD (20)
NA079	F	3y1m	10q24.31-q24.32	Chr10:102,953,588-103,040,185	Gain	87	2	*LBX1*	VSD	VSD (10)
NA008	F	2y8m	3q12.1- q12.2	Chr3:101,403,767-101,519,268	Gain	116	1	*TBC1D23*	VSD	/
NA082	F	3y1m	3p22.2	Chr3:37377757-37577933	Gain	201	3	*GOLGA4, C3ORF35, ITGA9*	ASD	/
			3q26.33-29	Chr3:180469228-198475603	Gain	18006	234	*SOX2, MAP3K13, BCL6, TP63, FGF12*	ASD	/
			4q34.3-q35.2	Chr4:181,026,511-190,784,769	Loss	9694	76	*CASP3, ING2, PDLIM3, SLC25A4, F11*	ASD	VSD (5)
NA084	F	1y2m	2q36.1	Chr2:222,835,872-223,511,548	Gain	676	6	*PAX3*	VSD	/
NA252	M	4 m	Xq13.3	ChrX:73900661-74462483	Gain	562	2	*KIAA2022,ABCB7*	VSD	/
			Xq21.1	ChrX:77,425,233-78,428,001	Loss	1003	11	*CYSLTR1, GPR174, LPAR4, P2RY10*	VSD	/
NA380	F	4y7m	16p13.11	Chr16:15,406,764-16,170,797	Gain	764	9	*MYH11, NDE1*	VSD	ASD (15), ASD (3)
NA423	M	5 m	16q24.1	Chr16:16,574,972-28,505,961	Loss	100	2	*ATP2C2, WFDC1*	VSD	/
NB1264	M	27d	4p16.1	Chr4:8,270,586-8,498,212	Loss	228	4	*ACOX3, C4orf23, HTRA3, SH3TC1*	VSD,ASD	/
NB245	M	1 m	16p13.11 - p11.2	Chr16:16,574,972-28,505,961	Gain	11931	135	*CACNG3, CHP2, PLK1, PRKCB, XYLT1*	VSD,ASD	ASD (15)
NB887	F	4 m	Xq22.1	ChrX:100,039,582-100,068,017	Gain	28	1	*XKRX*	VSD,ASD,PDA,PH	/
NB910	M	11d	13q13.3	Chr13:35,777,130-35,835,221	Gain	58	1	*NBEA*	VSD,PDA,PFO	/
			Xq27.2	ChrX:140,727,218-141,583,235	Gain	856	3	*MAGEC1, MAGEC2, MAGEC3*	VSD,PDA,PFO	/
NC15	M	5y	20p12.1	Chr20:16,574,972-28,505,961	Gain	120	1	*KIF16B*	VSD	ASD,VSD (15)
NC27	F	4y	15q13.1	Chr15:25,833,244-25,871,572	Loss	38	1	*OCA2*	VSD	/
NC28	M	3y	15q26.2	Chr15:92,616,792-92,673,355	Loss	57	1	*MCTP2*	VSD	AVSD, CHD (4), ASD
NS176	M	7y1m	7p14.2	Chr7:36657642-36756092	Gain	118	1	*AOAH*	VSD	ASD,VSD
		7y1m	14q32.12	Chr14:91069401-91230897	Gain	160	2	*C14orf184, CATSPERB*	VSD	ASD
NS480	M	4y	1q31.2	Chr1:190,543,305-190,707,353	Gain	164	1	*RGS21*	VSD	ASD (35)
NS494	M	2 m	2q14.2	Chr2:119,275,149-119,375,870	Gain	101	1	*EN1*	VSD,PFO,PH	ASD (5)
NS548	M	3y	7q11.22	Chr7:70,953,860-71,032,938	Loss	79	1	*CALN1*	VSD	VSD (15)
NS584	F	8 m	9q21.32	Chr9:84,859,691-85,387,778	Loss	528	2	*FRMD3, RASEF*	ASD,PS	/
NS659	F	12y5 m	7q31.32	Chr7:121,449,591-122,397,323	Gain	948	7	*AASS, CADPS2, FEZF1, PTPRZ1*	VSD	/
NS667	F	1y9m	21q22.3	Chr21:41639464-41733339	Gain	94	3	*FAM3B,MX2, MX1*	VSD	ASD (15)
NS8343	M	5 m	Xp22.2	ChrX:13,472,898-13,530,787	Loss	58	1	*EGFL6*	VSD,PDA,ASD,PFO	/

^a^Major genes means that they are not included all genes involved in the CNVs and the genes in bold are the candidate genes which have evidences derived from previous studies

^b^Reported phenotype in DECIPHER/ISCA/OMIM. Number in parenthesis is the number of features that the patient was affected. ASD, Atrial septal defect; VSD, Ventricular septal defect; CHD, congenital heart disease

### CNV comparison in isolated and complex VSD

We compared CNVs within the 100 isolated VSD patients with those 44 complex VSD patients (Additional file 1: Table S6). There was a trend towards increased CNV size in patients with complex VSD, but the difference did not reach statistical significance. There was no significant difference in rare CNV numbers (average CNV count for each case) for either deletions or duplications.

### Enrichment of CHD related genes

Several lines of evidence support the enrichment of CHD related genes within the CNVs detected in VSD patients. First, we found that *PAX3* and *LBX1* (in duplications) and *CRKL, GP1BB, PDLIM3, TBX1, TXNRD2* (in deletions) were annotated in the MGI database and CHD wiki as associated with CHD. Evidence from the literature and from GO signal pathway analysis further supported this notion (Tables 2 and 3). Second, the enrichment analysis revealed 25 genes of 285 genes within both duplication and deletion CNVs detected in this study enriched in transcription factor, chromatin binding and three of five biological processes associated with heart development or cardiovascular system development are the main functions for candidate genes (Table 4). Third, the top two networks constructed by IPA analysis for the 285 genes include networks of cardiovascular disease and network of herediary disorder (Score 46: 25 genes) (Fisher's exact test, *P* = 3.42E-08 to 3.79E-02) (Additional file 1: Table S7). Top transcription regulators (*NANOG, TP53, SOX2, POU5F1, IRF1*) inferred by IPA analysis were listed in the Additional file 1: Table S8 and Additional file 1: Figure S6C. As a homeobox, *NANOG* regulates several transcription factors [27] such as *EN1, SOX2, LBX1* and *ZFP42* in our dataset (*P* = 4.91E-03), which controls cellular growth, organic growth and development.

**Table 2:** Genes involved in CNVs related to in heart development

Data resource	Gene number	Hit no./total gene	Gene list for gain (172 genes) in our study	Gene list for loss (113 genes) in our study
MGI database	147	*7/285*	*PAX3, LBX1*	*CRKL* ^b^, *GP1BB* ^b^, *PDLIM3*, *TBX1* ^b^, *TXNRD2* ^b^
Candidate genes§	202	*9/285*	*PAX3, LBX1, MYH11, FGF12*	*CASP3, CRKL* ^b^, *PDLIM3, TBX1* ^b^, *TXNRD2* ^b^
Genes derived from GO:0072538 (*cardiovascular system development*)	1957	*9/285*	*PAX3, LBX1, MYH11, PRKCB, IGFBP7*	*CYSLTR1, LPAR4, CRKL* ^b^, *TBX1* ^b^, *TXNRD2* ^b^

^§^CHD wiki (47 genes), UCSC Genome Browser (104 genes), literatures (51genes); the overlapping genes between different datasets were merged. ^b^genes which were included in CNVs related to DiGeorge syndrome

**Table 3:** Genes involved in CNVs related to cell surface receptor signaling pathway and heart development

Known signal pathway^a^	Gene number	Hit no./total gene	Gene list for gain (172genes) in this study	Gene list for loss (113 genes) in this study
AKT pathway (VEGF, Insulin, MAPK, ErbB) (KEGG)	423	*5*	*CACNG3, CHP2, PRKCB*	*CASP3, CRKL* ^b^
FGF pathway (regulation of actin cytoskeleton) (KEGG)	212	*1*	*-*	*CRKL* ^b^
Hedgehog-Bmp pathway (KEGG)	56	*1*	*BMP5*	-
Notch pathway (Netpath)	100	*2*	*EN1*	*CASP3*
Notch pathway (KEGG)	44	0	-	-
TGF-BMP pathway (KEGG)	84	*1*	*BMP5*	-
Wnt pathway (Netpath)	121	*1*	*PRKCB*	-
Wnt pathway (KEGG)	264	*3*	*CACNG3,CHP2,PRKCB*	-
total	1304	*14/285*		

^a^CHD-related pathway from KEGG and Netpath, the overlapping genes between different datasets were merged

^b^genes which were included in CNVs related to DiGeorge syndrome

**Table 4:** Significantly enriched gene ontology (GO) terms from the genes involved in CNVs of VSD patients

	ID	Name	Genes	Genes input	Genes in Annotation	*P*-value
Molecular Function						
1	GO:0003682	chromatin binding	*HIRA,SOX2, PRKCB, ING2,TP63, BCL6,PAX3*	7	394	1.12E-04
2	GO:0043565	sequence-specific DNA binding	*PAX3, EN1, SOX2, TBX1, TP63, LBX1, BCL6*	7	741	7.04E-03
3	GO:0003700	sequence-specific DNA binding transcription factor activity	*HIRA, EN1, SOX2, PAX3, TBX1, TP63, LBX1, BCL6*	8	1052	8.35E-03
4	GO:0001071	nucleic acid binding transcription factor activity	*HIRA, EN1, SOX2, PAX3, TBX1, TP63, LBX1, BCL6*	8	1053	8.41E-03
Biological Process						
1	GO:0007507	heart development	*MYH11, TXNRD2, CRKL, FGF12, PDLIM3, TBX1, LBX1,CASP3,PAX3*	9	466	9.36E-06
2	GO:0072358	cardiovascular system development	*MYH11,TXNRD2,CRKL,FGF12,PDLIM3,TBX1,LBX1,CASP3, PRKCB,PAX3*	10	889	1.85E-04
3	GO:0072359	circulatory system development	*MYH11,TXNRD2,CRKL,FGF12,PDLIM3,TBX1,LBX1,CASP3, PRKCB,PAX3*	10	889	1.85E-04
4	GO:0042127	regulation of cell proliferation	*SOX2,CASP3,PAX3,BCL6,COMT,IGFBP7,LBX1,IL4R,TP63,TBX1,CHP2*	11	1338	1.21E-03
5	GO:0045596	negative regulation of cell differentiation	*SOX2, MED15, TBX1, TP63, LBX1, IL4R,BCL6*	7	527	7.52E-03
Cellular Component						
1	GO:0005667	transcription factor complex	*SOX2, PAX3, LBX1, ING2,TP63*	5	343	7.40E-03
2	GO:0044427	chromosomal part	*BCL6, PLK1, NDE1, ING1, TP63, HIRA*	6	596	1.00E-02
Mouse Phenotype						
1	MP:0003421	abnormal thyroid gland development	*PAX3, TBX1,CRKL*	3	14	1.14E-02
2	MP:0020135	abnormal heart ventricle thickness	*MYH11, TXNRD2, PAX3, LBX1, BCL6*	5	126	3.50E-02
3	MP:0006284	absent hypaxial muscle	*PAX3, LBX1*	2	3	3.71E-02
4	MP:0004914	absent ultimobranchial body	*PAX3, TBX1*	2	3	3.71E-02

*P*-value: Corrected by Bonferroni and cutoff is 0.05

## Discussion

Genomic imbalance, including known genomic disorders, contribute to the genetic etiology of congenital malformations such as CHD. In previous studies, syndromic chromosome abnormalities explained 6-9 % of CHD [28]. We found that Down syndrome (4 cases, 2.5 %), DiGeorge syndrome (2 cases, 1.2 %) and Trisomy X syndrome (2 cases, 1.2 %) contributed to up to 5 % of cases of VSD, consistent with the previous report [23, 24]. In addition, we identified large CNVs (> 1 Mb) (3/161, 1.9 %) including 4q34.3-q35.1, 3q26.32-q29 and 16p13.11-p11.2, which are associated with CHD as reported by DECIPHER and ISCA. Other CNV regions identified in our study such as 4q-ter, 15q26.2, and 16q22 had also been reported in the CHD wiki. We did not identify any significant difference in size, number or genic content of rare CNVs between complex VSDs and isolated VSDs. Some previous reports had reported a higher rate of CNVs carried in patients with CHD plus extracardiac or developmental abnormalities[5], but some claimed no significant increase[29]. We believe it likely that the genes affected by the CNVs are more important to cause VSD than CNV size or number, but the sample size might be too small to identify differences between isolated and complex VSDs.

Our interpretation suggests that critical genes contribute to the development of CHD by altered expression due to duplication or deletion CNVs. The genes identified in both *de novo* and recurrent CNVs were likely to be CHD-related genes. For example, we found a *de novo* deletion at Xp22.2 including *EGFL6. EGFL6* involved in the regulation of cell cycle, proliferation and developmental processes has been previously reported as a candidate gene for human developmental disorders and is expressed during embryonic development [30]. 16p13.11 duplication is recurrent in our cohort, it had also been reported to be significantly associated with CHD recently [31]. *MYH11* is the proposed candidate gene at this interval as defects in this gene underlie aortic aneurysm familial thoracic type 4 (AAT4) [MIM: 132900] and also contribute to familial thoracic aortic aneurysm and dissection (TAAD) and patent arterial duct (PDA). Our study suggests that *EGFL6* and *MYH11* may be dosage sensitive genes involved in embryonic heart development. Furthermore, we specifically evaluated genes involved in CNVs detected in patients with VSD. We identified 15 genes previously known to be associated with CHD or in CHD-related signal pathways (Tables 2 and 3). Among them, *CRKL, TBX1, TXNRD2, GP1BB* were known to be involved in DiGeorge syndrome. *MYH11, TXNRD2, PAX3, LBX1* and *BCL6* were associated with abnormal heart ventricle thickness (MP: 0020135). *BMP5, EN1, PRKCB, CACNG3* and *CHP2* were clustered in CHD related signaling pathways. Importantly, *CASP3, CRKL, FGF12, LBX1, MYH11, PDLIM3, TXNRD2* and *TBX1* are related to heart development (GO: 0007507) and also cardiovascular system development (GO: 0072358). It was inferred that these candidate genes might have effects on a wide range of cardiac tissues and regulate heart development at different stages.

Two types of molecular functions including chromatin binding and transcription factor complex were revealed through unbiased gene priority and enrichment analysis for all genes within CNVs of VSD patients and 5 biological processes via GO annotations, which indicated to be related to VSD. Transcription factors including *LBX1, PAX3, EN1, SOX2* and *TBX1* with confirmed effects on cardiogenesis were detected in our data set. *LBX1* is a homeodomian-containing transcription factor required for the diversification of heart precursor cells in *Drosophila* and its expression had been described in cardiac neural cells and in migrating muscle precursor cells [32]. The overexpression of *Lbx1* mRNA resulted in enlarged somites, an increase in cell proliferation by upregulating *MyoD* and lack of differentiated muscle [33]. *PAX3*, as a key regulatory factor in controlling the migrating of myogenic precursor cells, genetically acted in the upstream pathways of *Lbx1* and *Msx1. Pax3* also directly activate *MyoD* expression. The rising levels of *Pax3* and *Lbx1* result in enlarged muscle precursor cell population and then increase the bias for myogenic differentiation [34]. Additionally, a transcription regulation loop (*NANOG-SOX2-OTC4*) associated with downstream cascade regulation on *GATA4, NKX2.5, MESP* to modulate heart development (Additional file 1: Figure S6C). As the first formed organ, the genesis of heart involves a very complex series of morphogenetic interactions [35] and the transcription factors are essential for cardiogenesis at different embryonic stages.

As reported in the recent exome sequencing of CHD, *de novo* mutations in chromatin markers played a vital role in regulating cardiac development genes [36]. Seven genes (*HIRA, SOX2, PRKCB, ING2, TP63, BCL6* and *PAX3*) in this study were enriched in chromatin binding pathway (GO: 0003682) (*P* =1.12E-04)which are worthy of being investigated in more detail in future studies.

Based on our cohort, chromosomal imbalances account for 5.2 % (8/154) and rare CNVs account for 16.9 % (26/154) of the cases. No significant difference was detected in terms of CNV diagnostic yield between complex and isolated VSD patients, indicating that both populations should be tested for genomic imbalances. Although the VSD-related candidate genes (as shown in Table 5) need further studies to confirm their involvement in VSD pathogenesis, our findings demonstrated that high-density microarray analysis is a useful tool to uncover potential underline genomic causes for VSDs and extended enrichment and pathway analysis indicate possible convergence on pathways during cardiogenesis.

## Conclusions

In this pilot study, we identified genomic imbalances had an important contribution to the genetic burden of patient with VSD, which was consistent with the previous report in CHD. The rare CNVs VSD patients carried were interpreted and classified for clinical utility by comparing the population CNV database and patient-derived CNV database. CNV analysis of VSD patient in this study firstly showed genetic status of VSD on copy number variant and no significant difference between isolated VSD and complex VSD indicated that both populations need equal CNV tests. Furthermore, we applied gene enrichment and pathway analysis for understanding the relevant genes involved and the potential relevance of CNV with heart development, which may delineate the genetic etiology and pathways of VSDs.

**Table 5:** The 18 candidate genes for VSD identified in this study

Gene	Gene Annotation	CNV type	Hits into gene set	Phenotype (individual number)
*CRKL*	v-crk avian sarcoma virus CT10 oncogene homolog-like	loss	▪◆⋆△	iVSD (2) cVSD(1)
*LBX1*	ladybird homeobox 1	gain	▪◆△ο	iVSD
*PAX3*	paired box 3	gain	▪◆△ο	iVSD
*TBX1*	T-box 1	loss	▪◆△	iVSD (2) cVSD(1)
*PDLIM3*	PDZ and LIM domain 3	loss	▪◆	ASD
*TXNRD2*	thioredoxin reductase 2	loss	▪◆	iVSD (2) cVSD(1)
*GP1BB*	glycoprotein Ib (platelet), beta polypeptide	loss	◆△	iVSD (2) cVSD(1)
*CASP3*	caspase 3, apoptosis-related cysteine peptidase	loss	◆⋆△	ASD
*MYH11*	myosin, heavy chain 11, smooth muscle	gain	◆△ο	iVSD
*BMP5*	bone morphogenetic protein 5	gain	◆⊿△ο	iVSD
*EN1*	engrailed homeobox 1	gain	◆⊿△ο	cVSD
*PRKCB*	protein kinase C, beta	gain	◆⊿△ο	cVSD
*FGF12*	fibroblast growth factor 12	gain	◆ο	ASD
*HIRA*	histone cell cycle regulator	loss	⊿△ο	iVSD (2) cVSD(1)
*SOX2*	SRY (sex determining region Y)-box 2	gain	⊿ο	ASD
*DGCR2*	DiGeorge syndrome critical region gene 2	loss	⊿ο	iVSD (2) cVSD(1)
*PLK1*	polo-like kinase 1	gain	△ο	cVSD
*EGFL6*	EGF-like-domain, multiple 6	loss	△ *de novo*	cVSD

Note: ▪ MGI database, ◆ Genes within Geneset reported in literature and CHD wiki

⊿ Prioritized by Gene set from literature and CHD wiki

⋆Genes within GO and KEGG pathway

△ Prioritized by Gene set from GO and KEGG pathway

ο Genes significantly enriched by IPA

iVSD: isolated VSD; cVSD: complex VSD

## Additional file

Additional file 1:
**Supplemental material.** (DOCX 4259 kb)

## References

[1] Hoffman JI, Kaplan S (2002). The incidence of congenital heart disease. J Am Coll Cardiol.

[2] Gruber PJ, Epstein JA (2004). Development gone awry: congenital heart disease. Circ Res.

[3] Otterstad JE, Nitter-Hauge S, Myhre E (1983). Isolated ventricular septal defect in adults. Clinical and haemodynamic findings. Br Heart J.

[4] Hartman RJ, Rasmussen SA, Botto LD, Riehle-Colarusso T, Martin CL, Cragan JD (2011). The Contribution of Chromosomal Abnormalities to Congenital Heart Defects: A Population-Based Study. Pediatr Cardiol.

[5] Breckpot J, Thienpont B, Peeters H, de Ravel T, Singer A, Rayyan M (2010). Array comparative genomic hybridization as a diagnostic tool for syndromic heart defects. J Pediatr.

[6] van Karnebeek CDM, Hennekam RCM (1999). Associations Between Chromosomal Anomalies and Congenital Heart Defects: A Database Search. Am J Med Genet.

[7] Thienpont B, Mertens L, de Ravel T, Eyskens B, Boshoff D, Maas N (2007). Submicroscopic chromosomal imbalances detected by array-CGH are a frequent cause of congenital heart defects in selected patients. Eur Heart J.

[8] Greenway SC, Pereira AC, Lin JC, DePalma SR, Israel SJ, Mesquita SM (2009). De novo copy number variants identify new genes and loci in isolated sporadic tetralogy of Fallot. Nat Genet.

[9] Erdogan F, Larsen LA, Zhang L, Tumer Z, Tommerup N, Chen W (2008). High frequency of submicroscopic genomic aberrations detected by tiling path array comparative genome hybridisation in patients with isolated congenital heart disease. J Med Genet.

[10] Soemedi R, Wilson IJ, Bentham J, Darlay R, Topf A, Zelenika D (2012). Contribution of global rare copy-number variants to the risk of sporadic congenital heart disease. Am J Hum Genet.

[11] Obler D, Juraszek AL, Smoot LB, Natowicz MR (2008). Double outlet right ventricle: aetiologies and associations. J Med Genet.

[12] Prakash SK, LeMaire SA, Guo DC, Russell L, Regalado ES, Golabbakhsh H (2010). Rare copy number variants disrupt genes regulating vascular smooth muscle cell adhesion and contractility in sporadic thoracic aortic aneurysms and dissections. Am J Hum Genet.

[13] Geng J, Picker J, Zheng Z, Zhang X, Wang J, Hisama F (2014). Chromosome microarray testing for patients with congenital heart defects reveals novel disease causing loci and high diagnostic yield. BMC Genomics.

[14] Liao C, Li R, Fu F, Xie G, Zhang Y, Pan M (2014). Prenatal diagnosis of congenital heart defect by genome-wide high-resolution SNP array. Prenat Diagn.

[15] MacDonald JRZR, Yuen RK, Feuk L, Scherer SW (2014). The database of genomic variants: a curated collection of structural variation in the human genome. Nucleic Acids Res.

[16] Iafrate AJFL, Rivera MN, Listewnik ML, Donahoe PK, Qi Y, Scherer SW (2004). Detection of large-scale variation in the human genome. Nat Genet.

[17] Park H, Kim JI, Ju YS, Gokcumen O, Mills RE, Kim S (2010). Discovery of common Asian copy number variants using integrated high-resolution array CGH and massively parallel DNA sequencing. Nat Genet.

[18] Lin CH, Lin YC, Wu JY, Pan WH, Chen YT, Fann CS (2009). A genome-wide survey of copy number variations in Han Chinese residing in Taiwan. Genomics.

[19] Teo YY, Sim X, Ong RT, Tan AK, Chen J, Tantoso E (2009). Singapore Genome Variation Project: a haplotype map of three Southeast Asian populations. Genome Res.

[20] Teo YY SX, Ong RTH, Tan AKS, Chen JM, Tantoso E, Small KS (2009). Singapore Genome Variation Project: A Haplotype map of three South-East Asian populations. Genome Res.

[21] Firth HV, Richards SM, Bevan AP, Clayton S, Corpas M, Rajan D (2009). DECIPHER: Database of Chromosomal Imbalance and Phenotype in Humans Using Ensembl Resources. Am J Hum Genet.

[22] Chen J, Bardes EE, Aronow BJ, Jegga AG (2009). ToppGene Suite for gene list enrichment analysis and candidate gene prioritization. Nucleic Acids Res.

[23] Mihci E, Akcurin G, Eren E, Kardelen F, Akcurin S, Keser I (2010). Evaluation of congenital heart diseases and thyroid abnormalities in children with Down syndrome. Anadolu Kardiyol Derg.

[24] Tartaglia NR, Howell S, Sutherland A, Wilson R, Wilson L (2010). A review of trisomy X (47, XXX). Orphanet J Rare Dis.

[25] Rossi MR, DiMaio MS, Xiang B, Lu K, Kaymakcalan H, Seashore M (2009). Clinical and genomic characterization of distal duplications and deletions of chromosome 4q: study of two cases and review of the literature. Am J Med Genet A.

[26] Visser R, Gijsbers A, Ruivenkamp C, Karperien M, Reeser HM, Breuning MH (2010). Genome-wide SNP array analysis in patients with features of sotos syndrome. Horm Res Paediatr.

[27] Boyer LA, Lee TI, Cole MF, Johnstone SE, Levine SS, Zucker JP (2005). Core transcriptional regulatory circuitry in human embryonic stem cells. Cell.

[28] Tomita-Mitchell A, Mahnke DK, Struble CA, Tuffnell ME, Stamm KD, Hidestrand M (2012). Human gene copy number spectra analysis in congenital heart malformations. Physiol Genomics.

[29] Payne AR, Chang SW, Koenig SN, Zinn AR, Garg V (2012). Submicroscopic chromosomal copy number variations identified in children with hypoplastic left heart syndrome. Pediatr Cardiol.

[30] Buchner G, Orfanelli U, Quaderi N, Bassi MT, Andolfi G, Ballabio A (2000). Identification of a new EGF-repeat-containing gene from human Xp22: a candidate for developmental disorders. Genomics.

[31] Carey AS, Liang L, Edwards J, Brandt T, Mei H, Sharp AJ (2013). Effect of copy number variants on outcomes for infants with single ventricle heart defects. Circ Cardiovasc Genet.

[32] Jagla K, Frasch M, Jagla T, Dretzen G, Bellard F, Bellard M (1997). ladybird, a new component of the cardiogenic pathway in Drosophila required for diversification of heart precursors. Development.

[33] Martin BL, Harland RM (2006). A novel role for lbx1 in Xenopus hypaxial myogenesis. Development.

[34] Mennerich D, Braun T (2001). Activation of myogenesis by the homeobox gene Lbx1 requires cell proliferation. EMBO J.

[35] Dickinson DF, Arnold R, Wilkinson JL (1981). Ventricular septal defect in children born in Liverpool 1960 to 1969. Evaluation of natural course and surgical implications in an unselected population. Br Heart J.

[36] Zaidi S, Choi M, Wakimoto H, Ma L, Jiang J, Overton JD (2013). De novo mutations in histone-modifying genes in congenital heart disease. Nature.

