# Supplementary material for: Genome-wide copy number variant analysis for congenital ventricular septal defects in Chinese Han population
Source: BMC Med Genomics. 2016 Jan 8;9:2. doi: 10.1186/s12920-015-0163-4 (PMC4705616; doi:10.1186/s12920-015-0163-4)
Supplement: Additional file 1: — Supplemental material. (DOCX 4259 kb) [file 12920_2015_163_MOESM1_ESM.docx]

**Supplemental Material**

**Supplemental Material**

1. **Supplemental Tables**

Supplemental Table 1. Summary of clinical Information of our patients with VSD

Supplemental Table 2. MLPA probes used to validate the CNVs indentified by array CGH and to study parental samples (hg18)

Supplemental Table 3. CNV validation selected from the patients with family

Supplemental Table 4. CNV validation selected from the patients without samples of family

Supplemental Table 5. Summary of individuals with CNV size of >1Mb

Supplemental Table 6. Comparison of complex VSDs and isolated VSDs in population characteristics and CNV findings

Supplemental Table 7. The filtered 10 functions for cardiovascular disease by IPA

Supplemental Table 8. Top transcription regulators by IPA analysis

Supplemental Table 9. Thirty-two intergenic CNVs without gene involved

1. **Supplemental Figures**

Supplemental Figure 1. Classifications of VSD related MGI phenotype

Supplemental Figure 2. CHD related Pathway and Related Genes Selecting Process

Supplemental Figure 3. Percentage distribution of CNV frequencies for all CNVs and rare CNVs.

Supplemental Figure 4. Validation of selected de novo CNVs in NS8343 by MLPA.

Supplemental Figure 5. Ideogram summary of rare CNVs in 154 cases with VSDs.

Supplemental Figure 6. Molecular interaction of transcription regulators.

**Supplemental Table 1. Summary of clinical Information of our patients with VSD**

| sample ID | gender | Age  (y.m.d) | CHD phenotype | sample ID | gender | Age  (y.m.d) | CHD phenotype |
| --- | --- | --- | --- | --- | --- | --- | --- |
| Group A: Isolated VSD | | |  |  |  |  |  |
| NA007 | M | 1y3m | VSD | **NC23** | F | 5y | VSD |
| NA008 | M | 8m | VSD | **NC39** | F | 4y | VSD |
| NA012 | F | 1y8m | VSD | **NC57** | F | 37y | VSD |
| NA014 | M | 5m | VSD | **NC67** | M | 39y | VSD |
| NA015 | F | 2y6m | VSD | **NC-P2** | F | 10y | VSD |
| NA017 | M | 7m | VSD | **NC-P5** | F | 22y | VSD |
| NA021 | F | 4m | VSD | **NC-P8** | M | 3y | VSD |
| NA026 | F | 2m | VSD | **NC21** | F | 4y | VSD |
| NA027 | M | 1y3m | VSD | **NC27** | F | 4y | VSD |
| NA033 | M | 2m | VSD | **NC28** | M | 3y | VSD |
| NA043 | F | 1y4m | VSD | **NC32** | F | 11y | VSD |
| NA049 | M | 4m | VSD | **NC37** | M | 3y | VSD |
| NA050 | F | 2y5m | VSD | **NC49** | F | 8y | VSD |
| NA055 | M | 6m | VSD | **NC50** | M | 14y | VSD |
| NA065 | M | 1y3m | VSD | **NC52** | M | 18y | VSD |
| NA067 | M | 7m | VSD | **NC54** | M | 6y | VSD |
| NA070 | F | 2y8m | VSD | **NC69** | F | 24y | VSD |
| NA074 | F | 4m | VSD | **NC-P18** | F | 4y | VSD |
| NA075 | F | 2y8m | VSD | **NH25** | M | 4m | VSD |
| NA079 | F | 3y1m | VSD | **NH29** | M | 2y | VSD |
| NA084 | F | 1y2m | VSD | **NH41** | M | 2y | VSD |
| NA091 | F | 8m | VSD | **NH44** | M | 9m | VSD |
| NA092 | M | 1y8m | VSD | **NH53** | M | 1y | VSD |
| NA097 | F | 1y1m | VSD | **NS168** | M | 8y | VSD |
| NA101 | F | 1y | VSD | **NS176** | M | 7y1m | VSD |
| NA103 | F | 3m | VSD | **NS178** | M | 3y6m | VSD |
| NA107 | M | 1y5m | VSD | **NS232** | F | 3y | VSD |
| NA109 | M | 10y8m | VSD | **NS239** | F | 4y11m | VSD |
| NA110 | M | 11y3m | VSD | **NS250** | F | 12y | VSD |
| NA111 | F | 10y3m | VSD | **NS255** | F | 3y | VSD |
| NA112 | M | 7y1m | VSD | **NS348** | M | 10m | VSD |
| NA223 | M | 1m | VSD | **NS364** | M | 2y4m | VSD |
| NA236 | F | 5m | VSD | **NS392** | F | 3y8m | VSD |
| NA251 | M | 4m | VSD | **NS430** | F | 7y10m | VSD |
| NA252 | M | 4m | VSD | **NS443** | M | 2y5m | VSD |
| NA261 | M | 6m | VSD | **NS480** | M | 4y | VSD |
| NA380 | F | 4y7m | VSD | **NS502** | M | 6y4m | VSD |
| NA387 | M | 5m | VSD | **NS538** | F | 11y | VSD |
| NA397 | M | 6m | VSD | **NS539** | M | 2y7m | VSD |
| NA408 | M | 1y4m | VSD | **NS548** | M | 3y | VSD |
| NA410 | F | 1y11m | VSD | **NS549** | M | 13y2m | VSD |
| NA411 | F | 6m | VSD | **NS561** | F | 11y | VSD |
| NA412 | F | 3y5m | VSD | **NS568** | F | 2y | VSD |
| NA423 | M | 5m | VSD | **NS590** | F | 6y5m | VSD |
| NA434 | M | 11m | VSD | **NS608** | F | 3y6m | VSD |
| NB188 | M | 1m | VSD | **NS616** | M | 4y9m | VSD |
| NB295 | F | 13d | VSD | **NS621** | F | 4y3m | VSD |
| NB485 | M | 1m | VSD | **NS627** | F | 9y9m | VSD |
| NB753 | M | 1m | VSD | **NS641** | M | 4y1m | VSD |
| NB909 | M | 4m | VSD | **NS657** | F | 1y8m | VSD |
| NC14 | M | 3y | VSD | **NS659** | F | 12y5m | VSD |
| NC15 | M | 5y | VSD | **NS666** | F | 3y3m | VSD |
| Group B: Complex VSD | | |  |  |  |  |  |
| NA018 | F | 2y3m | VSD, PDA | **NB910** | M | 11d | VSD,PDA,PFO |
| NA019 | F | 5y | VSD, PDA | **NB921** | M | 2m | VSD,ASD |
| NA048 | M | 1y7m | VSD,ASD | **NC46** | F | 11y | VSD,EA |
| NA105 | M | 3y7m | VSD,ASD | **NC20** | F | 9y | VSD,ASD |
| NA185 | M | 5m | VSD,PDA | **NC31** | M | 20y | VSD,PH |
| NA360 | F | 2m | VSD,ASD,AS | **NC34** | F | 8y | VSD,PDA |
| NB1019 | M | 15d | VSD,ASD | **NC45** | M | 3y | VSD,PDA |
| NB1020 | M | 2m | VSD,ASD,PS,PDA | **NS397** | M | 1y6m | VSD,PH |
| NB1031 | M | 26d | VSD,ASD | **NS494** | M | 2m | VSD,PFO,PH |
| NB11 | M | 9m | VSD,EA | **NS532** | M | 4y | VSD,PH |
| NB1264 | M | 27d | VSD,ASD | **NS667** | F | 1y9m | VSD,PH |
| NB185 | F | 25d | VSD,PFO | **NS765** | M | 2m17d | VSD,PFO,PH |
| NB245 | M | 1m | VSD,ASD | **NS7786** | F | 7m | VSD,ASD |
| NB372 | F | 1m | VSD,ASD | **NS8159** | F | 11m | VSD,PFO,PH |
| NB406 | F | 1y | VSD,EA | **NS8207** | M | 8m | VSD,PFO |
| NB488 | F | 11m | VSD,PDA | **NS8221** | M | 2y5m | VSD,PDA,PFO |
| NB599 | M | 1m | VSD,ASD | **NS8222** | F | 7m | VSD,DORV,PFO,PH |
| NB666 | F | 1m | VSD,ASD | **NS8226** | M | 7m | VSD, PS |
| NB708 | M | 27d | VSD,PDA,PH | **NS8315** | F | 1m | VSD,ASD,PDA,MS |
| NB711 | F | 12d | VSD,PFO | **NS8343** | M | 5m | VSD,PDA,ASD,PFO |
| NB884 | M | 3m | VSD,ASD | **NS8775** | F | 9m | VSD,TGA |
| NB887 | F | 4m | VSD,ASD,PDA,PH |  |  |  |  |
| Group C: Other CHD | | |  |  |  |  |  |
| NA068 | F | 5y5m | TGA | **NB1286** | F | 1d | TOF |
| NA082 | M | 2m | ASD | **NC-P17** | F | 6y | PFO |
| NA114 | F | 1y6m | ASD | **NS584** | F | 8m | ASD, PS |
| NS7783 | M | 3m | ASD,TGA |  |  |  |  |

The abbreviation of phenotype used in this table : AS, Aortic stenosis；ASD, Atrial septal defect；PS, pulmonary stenosis；DORV, Double outlet of right ventricle；EA, Ebstein’s anomaly；PDA, Patent ductus anedosus；PFO, Patent foramen ovale；PH, Pulmonary hypertension；TGA, Transposition of great arteries；TOF, Tetralogy of Fallot；VSD, Ventricular septal defect. Age, age at diagnosis; F(Female), M (Male)

**Supplemental** **Table 2. MLPA probes used to validate the CNVs indentified by array CGH and to study parental samples (hg18)**

| **gene** | **MLPA probe sequence** |
| --- | --- |
| **AOAH-L** | GGGTTCCCTAAGGGTTGGAGTCTGTAATAGAACAGCTTGCTCAAG |
| **AOAH-R** | p-TTCACAACTCGACGGTCCAGGCCTCGTCTAGATTGGATCTTGCTGGCAC |
| **BMP5-L** | GGGTTCCCTAAGGGTTGGATGTGAATGGCATTTTCACCTGGAAAACAAGCGTCCTCT |
| **BMP5-R** | p-GCACCTCTCTTTATGCTGGATCTCTACGGATGCTAATCTAGATTGGATCTTGCTGGCAC |
| **BMPER-L** | GGGTTCCCTAAGGGTTGGACAAGCTCTGTGGTCTTTGTGGCAACTACAA |
| **BMPER-R** | p-TGGACATAAACGTGATGACTTAATTGGTGGAGCGAGTCTAGATTGGATCTTGCTGGCAC |
| **CATSPERB-L** | GGGTTCCCTAAGGGTTGGACGATTCTTCAGTGGACTCCTGGGGATGTGA |
| **CATSPERB-R** | p-TTCCAGAAAGTGAAATCAGTAAATTATATCCACATGTGGTCTAGATTGGATCTTGCTGGCAC |
| **CBARA1-L** | GGGTTCCCTAAGGGTTGGACTTACCAAAAGAGCATGCTTAGGTTAGG |
| **CBARA1-R** | p-GGGTCTGTGCCTCTACATACCTGAACGTCCGGATGCTGAAGTTCTAGATTGGATCTTGCTGGCAC |
| **CDK4-L** | GGGTTCCCTAAGGGTTGGATGCAGGTCGAAAAATGGGTGGATGGCCAAGGTAACCCTGGTGTTTGAGC |
| **CDK4-R** | p-ATGTAGACCAGGACCTAAGGACATATCTATGAATGACGCGACAGGAAGAACTTGATCTAGATTGGATCTTGCTGGCAC |
| **CYTL1-L** | GGGTTCCCTAAGGGTTGGATGTTGGAAAGTGGCCCAGGTAGATTCCTTG |
| **CYTL1-R** | p-AAGGACAAAGCACGGAAGCTGTACACCATCTCTAGATTGGATCTTGCTGGCAC |
| **EGFL6-L** | GGGTTCCCTAAGGGTTGGATGTGGGAGGCGAAAATTGGCCCTGGATGTAAGTTTGGTGAGTGCGTGG |
| **EGFL6-R** | p-GACCAAACAAATGCAGATGCTTTCCATGCTGCGTGTGGATGAGGCCATTCTAGATTGGATCTTGCTGGCAC |
| **EN1-L** | GGGTTCCCTAAGGGTTGGACGCCAAGATCAAGAAAGCCACA |
| **EN1-R** | p-GGCATCAAGAACGGCCTGGCGTGTGGGAGGCGAAAATTGGTCTAGATTGGATCTTGCTGGCAC |
| **FDX1-L** | GGGTTCCCTAAGGGTTGGATGTTGCTTTTGTCAGGTGCATGTGAGG |
| **FDX1-R** | p-GAACCCTGGCTTGTTCAACCTGTCACCTCATCTTTGATCTAGATTGGATCTTGCTGGCAC |
| **FGD3-L** | GGGTTCCCTAAGGGTTGGA AGCGAGCTCCAGGACCCATTGCTGC |
| **FGD3-L1** | GGGTTCCCTAAGGGTTGGAGTGTTCGATCCGAAAGGCTGGGCGCTGTTCCGTTCTCCAGGACCCATTGCTGC |
| **FGD3-R** | p-CCTAGGGATGCCAGACACTGGGCCTTCTGACCTTTCACATCTGGACAGCTCTAGATTGGATCTTGCTGGCAC |
| **FGD3-R1** | p-CCTAGGGATGCCAGACACTGGGCCTCAAAGCCGTCAAGGACAACGTGGATACCCGTCGTTCTAGATTGGATCTTGCTGGCAC |
| **FHIT-L** | GGGTTCCCTAAGGGTTGGATAAAAAACTACCGCAGTGGAGCGCTTCCATGACCTG |
| **FHIT-L1** | GGGTTCCCTAAGGGTTGGATAAAAAACTACCGCTTCTGGGAATTGCAGTCCCCGCTCTG |
| **FHIT-R** | p-CGTCCTGATGAAGTGGCCGATTTGTTTGAAAAGTCGGTGGTCTAGATTGGATCTTGCTGGCAC |
| **FHIT-R1** | p-CTCTGTCCGGTCACAGGACTTTTTGCATTCTCTGGTTTTCTAGATTGGATCTTGCTGGCAC |
| **FRMD3-L** | GGGTTCCCTAAGGGTTGGATCTGACCTTTCACATCTGGACAGCGCAGAGCCCACCAGTTGCTGAA |
| **FRMD3-R** | p-TTTAACTTGCTCCTGAAAGCTCACACGAACGTCCGGATGCTGAAGTTCTAGATTGGATCTTGCTGGCAC |
| **FUT5-L** | GGGTTCCCTAAGGGTTGGAAACGTCCGGATGCTGAAGTGATGGCAGAGCTACTGATCCTGCTGTGGACG |
| **FUT5-R** | p-TGGCCTTTTAACACACCCGTGAACGTCCGGATGCTGAAGTGATGGCAGAGTCTAGATTGGATCTTGCTGGCAC |
| **GRID2-L** | GGGTTCCCTAAGGGTTGGACTTAGCCTTTGGGCCAAAAGGTG |
| **GRID2-R** | p-GCAATAATCTTCTTATAACCTAAAACAACCTCTCTCCTCTAGATTGGATCTTGCTGGCAC |
| **KIF6-L** | GGGTTCCCTAAGGGTTGGATGTGAATGGGCACGTGATTTGGCAGATGGGTTTGTG |
| **KIF6-R** | p-AATAATAAGCGAGAAAGCTACAAATTTAAGTAAGTGTGAATGGGTCTAGATTGGATCTTGCTGGCAC |
| **LBX1-L** | GGGTTCCCTAAGGGTTGGATCATCCGGTGAAGAGATTCTGCCTCCGCCTGCCAACTCCAA |
| **LBX1-R** | p-CAAGCCACTGACGCCGTTCAGCATTCTGACCTTTCACATCTGGTCTAGATTGGATCTTGCTGGCAC |
| **LEPR-L** | GGGTTCCCTAAGGGTTGGATTAGGTCAGAGTCAGCCGGGAA |
| **LEPR-R** | p-GCCTTCTAAAGCGTGGCATACAGAGCGAGTCTAGATTGGATCTTGCTGGCAC |
| **MCTP2-L** | GGGTTCCCTAAGGGTTGGATCTGGACCCGTGATGGCTATGTGGAAGCAGTGACCTGAAT |
| **MCTP2-R** | p-GCTTCTATGACATCTCAACATTTTGAAGAACAATCTCATTCTCTGGTTTTCGTCTAGATTGGATCTTGCTGGCAC |
| **OSCAR-L** | GGGTTCCCTAAGGGTTGGAGGGGATGGGGGCCGGGTGAGGAAAGCTGGCTGATCTGGTGCTGATCCTCCAGCTGCT |
| **OSCAR-R** | p-GACCCTCTGTGAGTCACCCCTTCGATCCGAAAGGCTGGGCGCTGTTCCGTTCCTTTCTAGATTGGATCTTGCTGGCAC |
| **PAX3-L** | GGGTTCCCTAAGGGTTGGATCCCACAGTGTCCACTCCCCTC |
| **PAX3-R** | p-GGCCAGGGCCGCGTCAACCAGCTTCAGCGCAACACTCTAGATTGGATCTTGCTGGCAC |
| **PP8961-L** | GGGTTCCCTAAGGGTTGGACTAAATTAGTCAACCATGGCTGGGAGAC |
| **PP8961-R** | p-AGAGCCATGCAATCCAGACCCAGCTCTTTCTAGATTGGATCTTGCTGGCAC |
| **PTPRG-L** | GGGTTCCCTAAGGGTTGGATCGCCATCCTTCTGAAAGACGACTATTTTGTCAGTGGAGC |
| **PTPRG-R** | p-TGGTCTACCTGGCAGATTCAAAGCTGAGAAGGTGGAATTTTCTAGATTGGATCTTGCTGGCAC |
| **RASEF-L** | GGGTTCCCTAAGGGTTGGATCAAGCACACAACTTTTATTTGGTCTTTCAGCTCGAAGAACAAT |
| **RASEF-L** | GGGTTCCCTAAGGGTTGGAGAGCGGAAAGAGCATTATTCAGCGCCCGTGACGAAGCCAAGTTCATTCCC |
| **RASEF-R** | p-CAAAAGGAAGATGTGGCTGCATTGAAAAAACGAAAAGTCGGTGGTCTAGATTGGATCTTGCTGGCAC |
| **RASEF-R** | p-AGGTGCGAGTGTGAGCTGGGGAACGTCCGGATGCTGAAGTGATGGCAGAGTCTAGATTGGATCTTGCTGGCAC |
| **RLN1-L** | GGGTTCCCTAAGGGTTGGATACGTCCGGATGCTGAAGTGATGGCAGAGCCTGCTAGAATTCTGTTTACTACTG |
| **RLN1-R** | p-AACCAATTTTCCAGAGCAGTCGCGGTCGGTGAGACGTGGGAGGCGAAAATTGGCTCTAGATTGGATCTTGCTGGCAC |
| **SLC37A1-L** | GGGTTCCCTAAGGGTTGGAGAACGTCCGGATGCTGAAGACAGAGCCTTCATTTTTATTTTGACATTTCTG |
| **SLC37A1-R** | p-CTGTATGCAAGTTTTCACTTATCTCGTGTGGGAGGCGAAAATTGGTCTAGATTGGATCTTGCTGGCAC |
| **STK32B-L** | GGGTTCCCTAAGGGTTGGACCATTGGTAAAGGGAGTTTTGGAAAGGTAA |
| **STK32B-R** | p-GAATATAAATGTCTGGACCACTGGGCTTAACCTTATCTGGTTTCTAGATTGGATCTTGCTGGCAC |
| **UCHL3-L** | GGGTTCCCTAAGGGTTGGATGCCACGACGATGAACAGACGCCATCCGAGTTACTCATGAGACC |
| **UCHL3-R** | p-AGTGCCCATGAAGGTCAGACTGAGGAACGTCCGGATGCTGAAGTTCTAGATTGGATCTTGCTGGCAC |
| **WSCD1-L** | GGGTTCCCTAAGGGTTGGATCAGCGCAACACCTTGAGAAAGATGACTGTCTCCCACTGCCA |
| **WSCD1-R** | p-GGATGCGTGTGCTGAGCGGTGAGAACGTCCGGATGCTGAAGTTCTAGATTGGATCTTGCTGGCAC |
| **WSCD1-L1** | GGGTTCCCTAAGGGTTGGATCAGCGCAACACGATTGAGATGTTTGATTCAGCCATCCTGCT |
| **WSCD1-R1** | p-AATCCGGAACCCATACAGGTCCTGCTGCGTGTGGATGAGGCCTCTAGATTGGATCTTGCTGGCAC |
|  |  |

**Supplemental Table 3. CNV validation selected from the patients with family**

| Sample No. | start-end | CNV type | gene included | MLPA result | | | | | status |  |
| --- | --- | --- | --- | --- | --- | --- | --- | --- | --- | --- |
|  |  |  |  | proband | | paternal | | maternal |  |  |
| NS8159 | chr7:145,954,458-146,044,333 | loss | *CNTNAP2* | loss | loss | | normal | | inherited |  |
| NS8159 | chr6:155,667,715-155,950,767 | gain | *NOX3，TFB1M* | gain | gain | | normal | | inherited |  |
| NS532 | chr4:5020339-5290001 | gain | *CYTL1, SK3B* | gain | gain | | normal | | inherited |  |
| NS8343 | chrX:13484737-13527018 | loss | *EGFL6* | loss | normal | | normal | | *de novo* |  |
| NS176 | chr7:36657642-36756092 | gain | *AOAH* | gain | normal | | normal | | *de novo* |  |
| NS176 | chr14:91069401-91230897 | gain | *PP8961* | gain | normal | | normal | | *de novo* |  |
| NS608 | chr16:2823033-2922054 | Loss | *FLYWCN, PRSS30P, PRSS22* | loss | normal | | normal | | ND^a^ |  |
| NS608 | chr4:94102022-94207957 | Loss | *GRID2* | loss | normal | | normal | | ND^a^ |  |
| NS765 | chr20:14,570,643-14,654,563 | Loss | *MCROD2* | Loss | normal | | normal | | ND^a^ |  |
| NS765 | chr2:242,661,265-242,951,149 | Loss | *LOC728323* | Loss | normal | | normal | | ND^a^ |  |
| NS430 | chr11:109807662-109924635 | gain | *FDX1* | gain | normal | | gain | | inherited |  |
| NS568 | chr17:5960595-6002116 | gain | *WSCD1* | gain | normal | | gain | | inherited |  |
| NS8221 | chr10:73,849,899-73,981,126 | loss | *CBARA1* | loss | loss | | normal | | inherited |  |
| NS7866 | chr7:34091981-34140619 | gain | *BMPER* | normal | normal | | normal | | no |  |
| Note: ND^a^, stepparents detected by paternity test, not determined; Inheritance, whether or not the CNV was found in a parent based on MLPA. no, failed to confirm. | | | | | | |  | |  |  |

|  |  |  |
| --- | --- | --- |

**Supplemental Table 4. CNV validation selected from the patients with no parental samples**

| Sample No. | start-end | CNV type | gene included | MLPA result |
| --- | --- | --- | --- | --- |
| NA380 | chr16:15,406,764-16,170,797 | gain | *MYHI1, NDE1E* | yes |
| NS667 | chr21:41639464-41733339 | gain | *MX1, FAM3B, MX2* | yes |
| NA043 | chr8:71820190-72802278 | gain | *EYA1* | yes |
|  | chr6:39,676,620-39,817,960 | gain | *KIF6* | yes |
| NA185 | chr20:60,516,672-60,588,679 | loss | *Mir-133, Mir-1* | yes |
| NS659 | chr2:140,698,923-141,017,430 | gain | *LRP1B* | yes |
| NB708 | chrX:126,961,245-127,062,292 | loss | *ACTRT1* | yes |
| NS584 | chr9:84866206-85378735 | loss | *RASEF, FRMD3* | yes |
| NS494 | chr2:119275149-119375870 | gain | *EN1* | yes |
| NA012 | chr6:55823840-55949133 | gain | *BMP5* | yes |
| NA079 | chr10:102,953,588-103,040,185 | gain | *LBX1* | yes |
| NC-P17 | chr13:75044367-75146038 | gain | *UCHL3* | yes |
| NC28 | chr15:92616792-92673355 | loss | *MCTP2* | yes |
| NC31 | chr21:42702390-42886269 | gain | *SLC37A1* | yes |
| NC39 | chr19:5789002-5818730 | loss | *FUT5* | ND^b^ |
| NC52 | chr12:56429479-56460713 | gain | *CDK4* | yes |
| NB21 | chr19:59261286-59321465 | gain | *OSCAR* | yes |
| NA084 | chr2:222826456-223507433 | gain | *PAX3* | yes |
| NA411 | chr1:65707463-65770735 | gain | *LEPR* | no |
| NA049 | chr1:65696259-65796571 | gain | *LEPR* | no |
| NS8159 | chr1:65696259-65770535 | gain | *LEPR* | no |
| NS397 | chr4:94,102,022-94,207,957 | loss | *GRID2* | yes |
| NS608 | chr4:94,102,022-94,207,957 | loss | *GRID2* | yes |
| NS8221 | chr10:73,849,899-73,981,126 | loss | *CBARA1* | yes |
| NB1031 | chr10:73,849,899-73,981,126 | loss | *CBARA1* | yes |
| Note: ND, not determined；no , failed to confirm.; ND^b^, probes failed to work | | | |  |
|  | | | |  |

**Supplemental Table 5. Summary of individuals with CNV size of >1Mb**

| Case No. | phenotype | gender | Length(Mb) | type | cytoband | disease |
| --- | --- | --- | --- | --- | --- | --- |
| NS255 | VSD | Female | 2.83 | loss | 22q11.2 | 22q11.2 deletion. |
| NS7783 | VSD,TGA | Male | 2.69 | loss | 22q11.2 | 22q11.2 deletion. |
| NB245 | VSD,ASD | Male | 11.9 | gain | 16p13.11-p11.2 | 16p12 duplication |
| NA82 | ASD,PFO | Male | 10 | loss | 4q34.3-q35.1 | 4q deletion syndrome |
|  |  |  | 18 | gain | 3q26.32-q29 | 3qter duplication |
| NA252 | VSD | Male | 1 | Loss | Xq21.1 | novel |

The abbreviation of phenotype used in this table : ASD, Atrial septal defect；PFO, Patent foramen ovale； TGA, Transposition of great arteries；VSD, Ventricular septal defect.

**Supplemental Table 6. Comparison of complex VSDs and isolated VSDs in population characteristics and CNV findings**

|  | **iVSD N（%）** | **cVSD**  **N（%）** | | **P value** |
| --- | --- | --- | --- | --- |
| **Number of Samples** | 102 | 46 | |  |
| Trisomy X/Down sydnrome | 2 | 3 | |  |
| **Basic information of patients with VSDs** | | | |  |
| Gender |  |  | | 0.40 |
| Male | 53(52.0%) | 27 (58.6%) | |  |
| Female | 49(48.0%) | 20 (43.2%) | |  |
| Age |  |  | |  |
| ≦1 yo | 30(29.4%) | 32(69.5%) | | 0.00 |
| >1 yo | 72(70.6%) | 15(32.6%) | |  |
| **Information of patients with rare CNVs** | | | |  |
| Gender |  | |  | 1.0 |
| Male | 12(75.0%) | | 5(71.4%） |  |
| Female | 4(25.0%） | | 2(28.6%） |  |
| Age |  | |  | 0.005 |
| ≦1 yo | 3(18.8%) | | 6(85.7%) |  |
| >1 yo | 13(81.3%) | | 1(14.3%) |  |
| **Rare CNV events** |  | |  | 0.63 |
| individuals without rare CNVs | 83(83.0%) | | 39(86.7%) |  |
| individuals with rare CNVs | 16(17.0%) | | 7(13.3%) |  |
| **CNVs type** |  | |  | 1.0 |
| Gain | 14(77.8%) | | 6(85.7%) |  |
| Loss | 4(22.2%) | | 1(14.4%) |  |
| **Count of rare CNVs**  (Count of CNVs per individual) | 18(1.1) | | 7(1.0) |  |
| **CNV size*** |  | |  |  |
| <**5**00kb | 12(66.7%) | | 4(57.1%) | 0.67 |
| >500kb | 6(33.3%) | | 3(42.9%) |  |
| CNV(kb) (max) | 1002.77 | | 11930 |  |
| CNV(kb)(min) | 40.89 | | 28.44 |  |
| CNV(kb)(mean) | 319.5±331.7 | | 2178.0±4788.3 | 0.38 |
| **Gene covered** (mean)***** | 2.6±2.5 | | 16.4±39.1 | 0.39 |

Abbreviations: iVSD, isolated VSD; cVSD, complex VSD.

Note: *, two cases with DiGeorge syndrome were excluded; P value is based on the two-tailed Fisher exact test and student T-test.

**Supplemental Table 7. The filtered eight functions for cardiovascular disease by IPA**

| **category** | **Functions Annotation** | **Molecules** | **p-Value** |
| --- | --- | --- | --- |
| Cardiovascular Disease | DiGenorge syndrome | *↓DGCR8, ↓GP1BB, ↓TBX1* | 3.42E-08 |
| Cardiovascular Disease | Shprintzen syndrome (VCFS) | *↓GP1BB, ↓TBX1* | 9.20E-05 |
| Cardiovascular Disease | Liddle syndrome | *↑SCNN1B, ↑SCNN1G* | 2.74E-04 |
| Cardiovascular Disease | Hyperplasia of heart | *↑LBX1,* | 4.78E-03 |
| Cardiovascular Disease | Hyperplasia of myocardium | *↑LBX1* | 9.62E-03 |
| Cardiovascular Disease | Fibrosis of myocardium | *↑PRKCB* | 1.41E-02 |
| Cardiovascular Disease | Overriding aorta | *↓CRKL, ↓TBX1* | 2.21E-02 |
| Cardiovascular Disease | Dilated cardiomyopathy | *↓DGCR8, ↑MYH11, ↓XYLT1* | 3.70E-02 |

Note: The score is based on a p-value calculation, which calculates the likelihood that the Network Eligible Molecules that are part of a network are found therein by random chance alone. Mathematically, the score is simply the negative exponent of the right-tailed Fisher's exact test result. ↑gain, ↓loss.

**Supplemental Table 8. Top transcription regulators by IPA analysis**

| **Transcription Regulator** | **Target molecules in dataset** | **p-value of overlap** |
| --- | --- | --- |
| NANOG | *↖EN1, ↖LBX1, ↑SOX2, ↓ZFP42* | 4.91E-03 |
| TP53 | *↑ABCC1, ↑ACSL3, ↓CASP3, ↓EGFL6, ↑IGFBP7, ↓KLKB1, ↑POLR3E* | 5.88E-03 |
| SOX2 | *↑ABCC6, ↓EN1, ↖LBX1, ↓ZFP42* | 6.33E-03 |
| POU5F1 | *↖EN1, ↖LBX1, ↑SOX2, ↓ZFP42* | 8.76E-03 |
| KLF4 | *↖MYH11, ↑PAX3, ↑SOX2* | 2.07E-02 |
| IRF1 | *↘CASP3, ↑IL27, ↓IRF2, ↑MX1, ↓TLR3* | 1.18E-02 |

Note: ↑activated, gain in our dataset, ↖regulated, gain in our dataset, ↓inactivated, loss in our dataset, ↘ regulated, loss in our dataset; p-value is overlap p-value measure which is calculated using Fisher exact test.

**Supplemental Table 9. Thirty-two intergenic CNVs without gene involved**

| Sample | Interval | CN | Size(bp) | Cytoband | DGV record＆ | Non-coding RNA |
| --- | --- | --- | --- | --- | --- | --- |
| NA007 | chr2:52,173,191-52,346,323 | CN Loss | 173133 | p16.3 | 0 |  |
| NA008 | chr4:157,466,503-157,652,012 | CN Loss | 185510 | q32.1 | 0 |  |
| NA097 | chrX:75,924,358-76,023,277 | CN Loss | 98920 | q13.3 - q21.1 | 0 | Mir325HG |
| NA261 | chr5:0-140,224 | CN Gain | 140225 | p15.33 | 1 |  |
| NA261 | chr4:18,544,391-18,865,674 | CN Loss | 321284 | p15.31 | 0 |  |
| NB1020 | chr8:138,210,323-138,575,475 | CN Loss | 365153 | q24.23 | 1 |  |
| NB185 | chr4:130,938,545-131,105,462 | CN Gain | 166918 | q28.2 | 1 |  |
| NB245 | chr5:0-140,224 | CN Gain | 140225 | p15.33 | 1 |  |
| NB406 | chrX:13,360,705-13,472,898 | CN Gain | 112194 | p22.2 | 1 |  |
| NB599 | chr3:0-189,305 | CN Gain | 189306 | p26.3 | 1 |  |
| NB599 | chr5:0-157,564 | CN Gain | 157565 | p15.33 | Han |  |
| NB599 | chr9:11,876,416-12,339,777 | CN Loss | 463362 | p23 | 1 |  |
| NB599 | chr3:0-189,305 | CN Gain | 189306 | p26.3 | 1 | LOC102723448 |
| NB910 | chr4:18,651,610-18,865,674 | CN Loss | 214065 | p15.31 | Han |  |
| NC15 | chrX:93,250,735-94,232,251 | CN Gain | 981517 | q21.32 - q21.33 | 1 |  |
| NC20 | chr3:5,758,138-6,241,569 | CN Gain | 483432 | p26.1 | 1 |  |
| NC23 | chr7:3,102,313-3,186,606 | CN Loss | 84294 | p22.2 | 1 |  |
| NC50 | chr14:41,940,157-42,037,333 | CN Loss | 97177 | q21.2 | 0 |  |
| NC54 | chr3:553,118-841,385 | CN Loss | 288268 | p26.3 | 1 | LINC01266 |
| NCHD | chr7:3,102,313-3,298,515 | CN Gain | 196203 | p22.2 | 1 | LOC100129603 |
| NH41 | chr1:105,828,253-106,085,353 | CN Loss | 257101 | p21.1 | 1 |  |
| NH53 | chr6:142,177,894-142,283,819 | CN Loss | 105926 | q24.1 | 1 |  |
| NS250 | chr16:62,242,802-62,448,002 | CN Gain | 205201 | q21 | 0 |  |
| NS250 | chr6:54,380,036-54,616,676 | CN Loss | 236641 | p12.1 | 0 |  |
| NS250 | chr16:62,242,802-62,448,002 | CN Gain | 205201 | q21 | 0 |  |
| NS364 | chr13:89,831,433-90,285,596 | CN Gain | 454164 | q31.3 | 0 | LINC00440,LINC01040,  LINC0350 |
| NS621 | chr15:18,362,555-18,555,748 | CN Loss | 193194 | q11.1 - q11.2 | 0 |  |
| NS666 | chr6:95,494,030-95,681,651 | CN Loss | 187622 | q16.1 | 1 |  |
| NS667 | chr15:18,362,555-18,555,748 | CN Loss | 193194 | q11.1 - q11.2 | 0 |  |
| NS765 | chr14:41,940,157-42,037,333 | CN Loss | 97177 | q21.2 | 0 |  |
| NS8222 | chr16:62,940,196-63,444,409 | CN Loss | 504214 | q21 | 0 |  |
| NS8226 | chr13:64,886,790-65,057,876 | CN Loss | 171087 | q21.32 | 0 |  |

＆: It indicated if it is included in the DGV database. 1, yes; 0, no. If it was involved in Han population database from healthy control, it shows “han”.


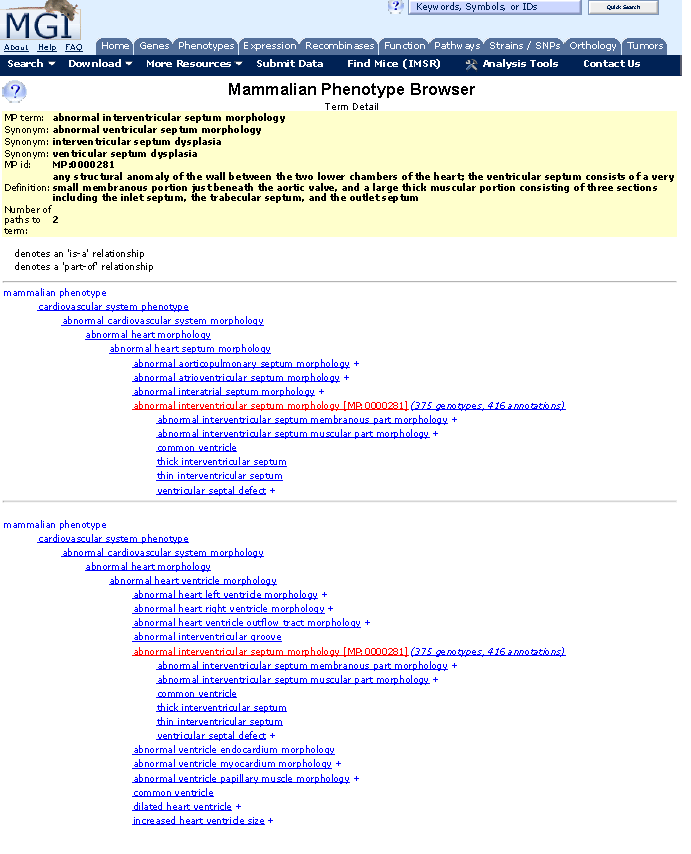


**Supplemental Figure 1. Classifications of VSD related MGI phenotype**

Mouse Genome Informatics resource (MGI, <http://www.informatics.jax.org/>) with VSD was applied to select phenotype-related gene lists. This term is defined as four parts, which are any structural anomaly of the wall between the two lower chambers of the heart, the ventricular septum consists of a very small membranous portion just beneath the aortic valve, and a large thick muscular portion consisting of three sections including the inlet septum, the trabecular septum, and the outlet septum. We found 147 genes with 375 genotype and 416 annotations related to “abnormal interventricular septum morphology” which covered “abnormal interventricular septum membranous part morphology”, “abnormal interventricular septum muscular part morphology and muscular part morphology”, “common ventricle”, “[thick interventricular septum](http://www.informatics.jax.org/searches/Phat.cgi?id=MP:0010724)”, “thin interventricular septum” , “[ventricular septal defect](http://www.informatics.jax.org/searches/Phat.cgi?id=MP:0010402)”.


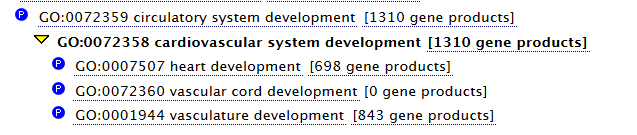


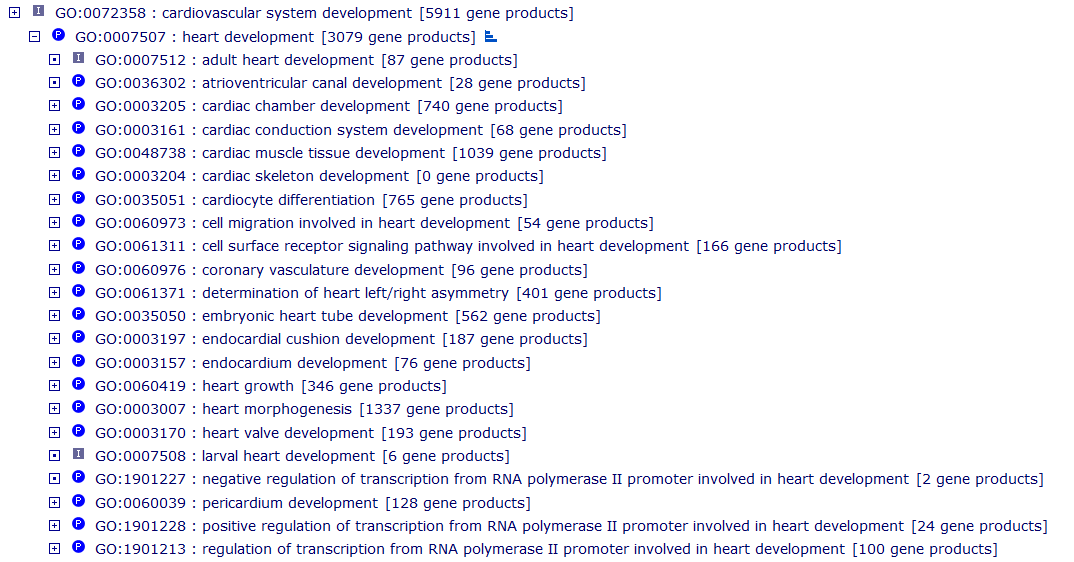


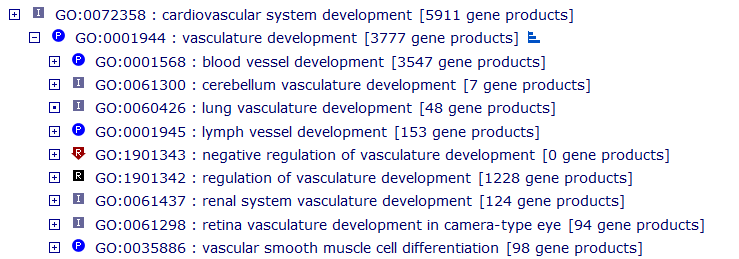


**Supplemental Figure 2. CHD related Pathway and Related Genes Selecting Process**

For Go annotations of CHD related genes, we focus on *cardiovascular system development* (GO:00072538). The subgroups of GO:00072538 are *heart development* (GO: 0007507) and *vasculature development* (GO:0001944). There are almost 1957 genes involved in these pathways as dataset for further analysis. Alternatively, we notice *cell surface receptor signaling pathway involved in heart development* (GO:0061311) whose descendants available are *fibroblast growth factor receptor signal pathway involved in heart development* (GO:0061313), *Notch signaling pathway* (GO:0061314), *BMP signaling pathway* (GO:0061312), *smoothened receptor signaling pathway involved in regulation of secondary heart field cardioblast proliferation*(GO:0003271), *Wnt receptor signaling pathway* (GO:0003306), *transforming growth factor beta receptor signaling pathway* (GO:00003302). Also we complemented the genes of above pathways and other related signaling pathway such as AKT and Hedghog pathway from KEGG and Netpath. After removing the genes which is not exist in human being, we got 611 genes of CHD-related GO signal pathway. Red line described the GO annotations we selected.

**Supplemental Figure 3. Percentage distribution of CNV frequencies for all CNVs and rare CNVs.** We described the distribution of CNV size by categorizing it three groups ( <100kb, 100-500kb, and >500kb).


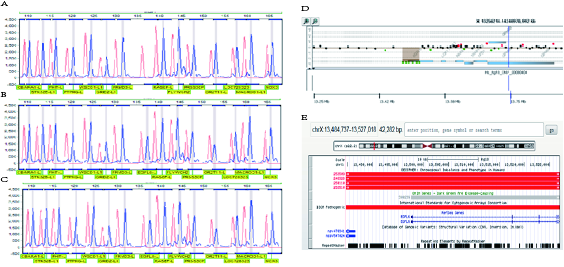


E


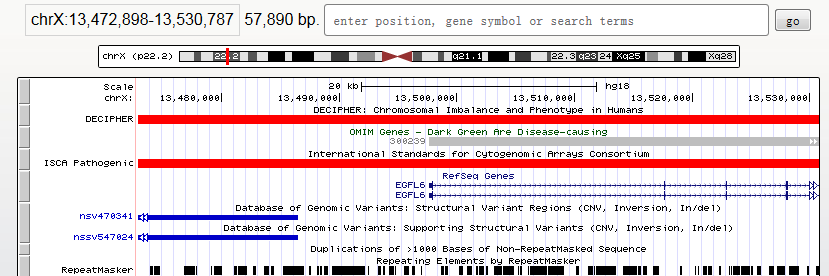


D


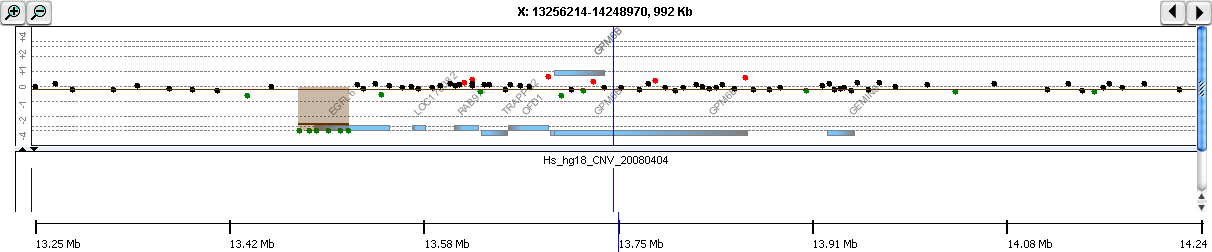


**Supplemental Figure 4. Validation of selected *de novo* CNVs in NS8343 by MLPA.**

1. Peak pattern of loci-specific design MLPA test obtained with proband of NS8343, black arrow showed the deletion of *EGFL6*; B) Peak pattern of loci-specific design MLPA test obtained with father of NS8343, black arrow showed normal copy of *EGFL6*; C) Peak pattern of loci-specific design MLPA test obtained with mother of NS8343, black arrow showed normal copy of *EGFL6*; D) 57kb loss in *EGFL6* in proband of NS8343 detected by Array CGH 244k; E) The customer track of chrX:13,472,898-13,530,787 by UCSC browse.


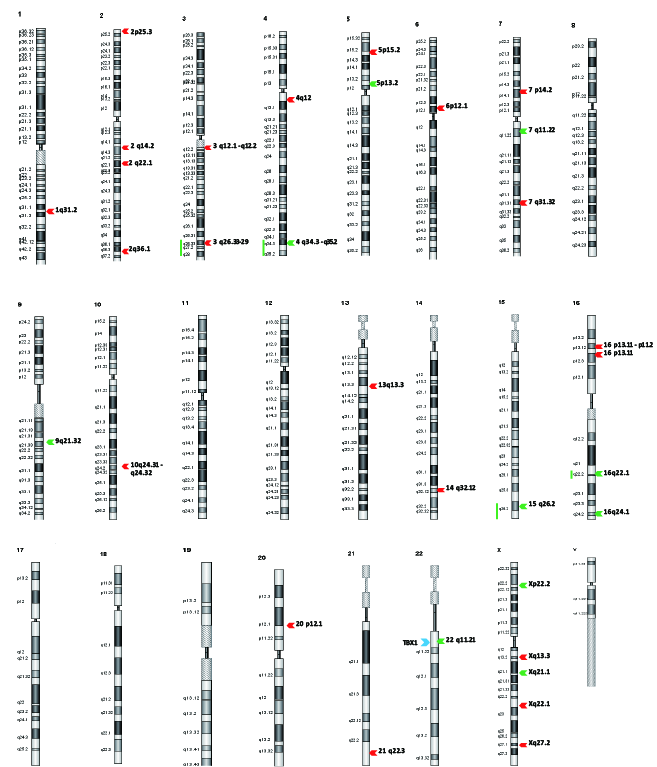


**Supplemental Figure 5. Ideogram summary of rare CNVs in 153 cases with VSDs.** Red and green indicate loss and gain in copy numbers, respectively. The vertical bars represent for loss/gain derived from CHD wiki. The blue arrow shows the known candidate genes of CHD. There is no consideration for the variants in Y chromosome.

A-B


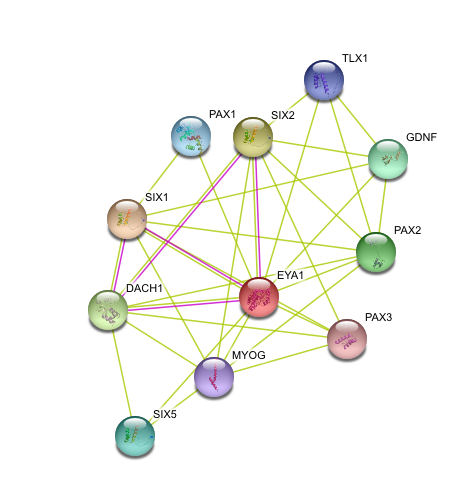

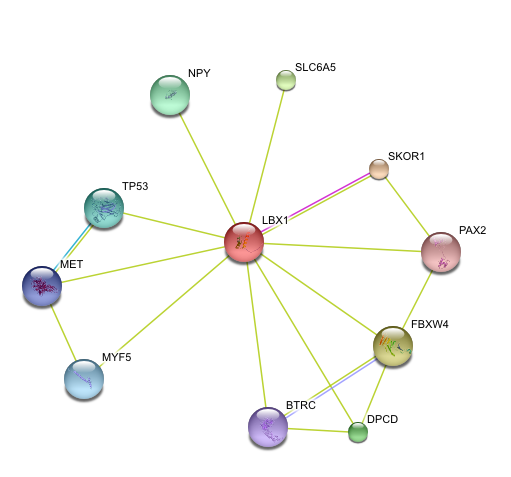


Cdf


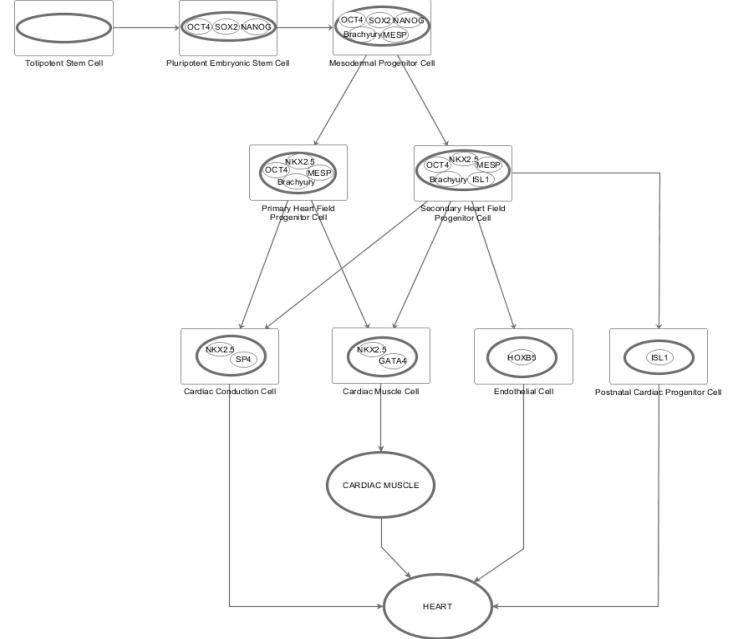


**Supplemental Figure S6. Molecular interaction of transcription regulators by IPS.**

A-B) The physical interactions between LBX1 and other protein analyzed by STRING;

C) NANOG-SOX2-OTRC4 member as transcription regulator regulates the downstream transcription factors to involve in embryonic stem cell differentiations into cardiac lineages
